# Hinged-3D metamaterials with giant and strain-independent Poisson’s ratios

**DOI:** 10.1038/s41598-020-59205-x

**Published:** 2020-02-10

**Authors:** Mohamed Shaat, Ahmed Wagih

**Affiliations:** 1 Mechanical Engineering Department, Abu Dhabi University, P.O.BOX 1790, Al Ain, United Arab Emirates; 2 Department of Mechanical Engineering, Zagazig University, Zagazig, 44511 Egypt

**Keywords:** Materials science, Mechanical engineering, Design, synthesis and processing

## Abstract

Current designs of artificial metamaterials with giant Poisson’s ratios proposed microlattices that secrete the transverse displacement nonlinearly varies with the longitudinal displacement, and the Poisson’s ratio depends on the applied strain (i.e., tailorable Poisson’s ratio). Whereas metamaterials with tailorable Poisson’s ratios would find many important applications, the design of a metamaterial with a giant Poisson’s ratio that is constant over all the material deformation range has been a major challenge. Here, we develop a new class of bimaterial-3D-metamaterials with giant and strain-independent Poisson’s ratios (i.e., Poisson’s ratio is constant over the entire deformation range). The unit cell is 3D assembled of hinged-struts. Specially designed spherical hinges were utilized to give constant Poisson’s ratios. This new class of metamaterials has been demonstrated by means of experimental and numerical mechanics. 15 material samples were 3D printed by Stereolithography (SLA) and tested. We revealed a robust anisotropy dependence of the Poisson’s ratio. A giant negative Poisson’s ratio of −16 was obtained utilizing a highly anisotropic unit cell of dissimilar materials and stiffnesses. Materials with giant and strain-independent Poisson’s ratios provide a new class of artificial metamaterials, which would be used to optimize the performance of many existing devices, e.g., strain amplifiers and gauges.

## Introduction

Artificial metamaterials are multiscale materials with exceptional macroscopic behaviors arising from the design of their microstructures beyond those of conventional materials found in nature^1,2^. The microstructure of a metamaterial can be tailored and optimized to promote the activation of nontraditional microscopic phenomena^2,3^. When these phenomena are activated, a metamaterial exhibits extraordinary behaviors beyond those observed in conventional materials. For example, membrane-type acoustic metamaterials with weak elastic moduli can give low-frequency oscillation patterns, which would produce an equivalent negative mass density of sound attenuation in specific frequency ranges^4–6^. In addition, metamaterials of cubic symmetric 3D-unit cells with special topologies that promote microstructural buckling can give auxetic behaviors; i.e., an auxetic metamaterial is a material with a negative Poisson’s ratio^7^. Furthermore, metamaterials with crosslinked-3D unit cells can be fabricated to give an equivalent negative static compressibility^8^. By tailoring the mechanism of interaction between the inclusions and the matrix, composite metamaterials with equivalent negative mass densities, moduli, and/or Poisson’s ratios can be obtained^2,9–12^. A unit cell of hexagonal sub-units has been proposed to give a mechanical metamaterial with negative stiffness and negative Poisson’s ratio^13^. Other approaches that have been used for making metamaterials with negative Poisson’s ratios depended on hierarchical^14^, chiral^15,16^, kirigami^17,18^, and lattice^19,20^ unit cells. Recently, mechanical metamaterials that exhibit non-reciprocity were designed (natural materials are reciprocal materials, which elastically deform symmetrically when the applied load direction is switched)^21^.

A new horizon of advanced materials that has come to the fore recently is metamaterials with giant Poisson’s ratios. The microlattices of these materials are specially designed to give Poisson’s ratios beyond those of natural materials. The Poisson’s ratio (*ν*) of a natural material is typically within the range $0 < \nu  < 0.5$. However, an early study revealed a giant Poisson’s ratio of −12 for a microporous foam of an open network of crosslinked-anisotropic disc-shaped particles of expanded polytetrafluoroethylene^22^. This microstructure was designed to activate micro-rotation fields, which led to the measured giant Poisson’s ratio. In addition, early investigations on the Poisson’s ratio of FCC and BCC cubic crystals revealed a Poisson’s ratio within the range $-1 < \nu  < 2$, which were attributed to the contrast between the interatomic forces along the different crystal directions^23,24^. In a recent study, it was demonstrated that nano-interstitials would give auxetic FCC crystals of giant Poisson’s ratios^25^. Lattice metamaterials with sinusoid-shaped beams gave high Poisson’s ratios that vary between −0.7 and 0.5 as functions of the applied strain^26^. 3D architected lattice system with curved beams was used to propose metamaterials with negative Poisson’s ratios that vary with the applied strain from −1 to 0^20^. It was revealed that carbon nanotubes can secrete materials with giant positive Poisson’s ratios. Yarns made of multi-walled carbon nanotubes gave a giant Poisson’s ratio of 4.2^27^, and carbon nanotube aerogel sheets achieved a giant Poisson’s ratio of 15^28^. Slabs made of fiber networks reflected a giant auxetic behavior with −5.7 Poisson’s ratio. Molecular dynamics simulations demonstrated giant negative Poisson’s ratios of graphene ribbons of −1.51^29,30^ and corrugated graphene sheets of −10^31^. Recent studies demonstrated giant negative Poisson’s ratios of artificial metamaterials with microlattices of dissimilar materials^32^. For instance, 3D printed-multimodulus metamaterials gave −7 Poisson’s ratio^32,33^.

Whereas previous studies proposed metamaterials with giant Poisson’s ratios^22,27–29,32,33^, the Poisson’s ratio being tailorable as the applied strain increases is a must. For example, the Poisson’s ratio of microporous foam varied between ∼1 and −12 when increasing the applied strain from 2% to 35%^22^. Although materials with tailorable Poisson’s ratio would find some important applications, the wider range of applications requires the material maintains its Poisson’s ratio over a wide range of strains. A material with a tailorable Poisson’s ratio is preferred to be implemented over a specific strain range. Outside this range, the material is conventional. A material with strain-dependent Poisson’s ratio is not preferred in some application, e.g., strain amplification. Metamaterials with giant negative Poisson’s ratios were proposed as strain amplifiers^34^. An efficient strain amplifier has a constant amplification factor - that is bigger than −1 - for the different input strains. Therefore, materials with strain-independent (i.e., constant) and giant Poisson’s ratios are required for making strain amplifiers. Generally speaking, for the material design and implementation purposes, the Poisson’s ratio would be required to be constant over a wide range of applied strains.

Here, we develop a new class of hinged-3D metamaterials with giant and strain-independent Poisson’s ratio. The Poisson’s ratio is constant over the different material deformation patterns and deformation ranges. The Poisson’s ratio of our developed metamaterial only depends on the material composition and geometry of its unit cell. The new class of metamaterials developed here will find a wide range of applications that require materials with constant properties over a wide range of the material deformation, e.g., strain amplifiers.

## Results

**Figure 1:**
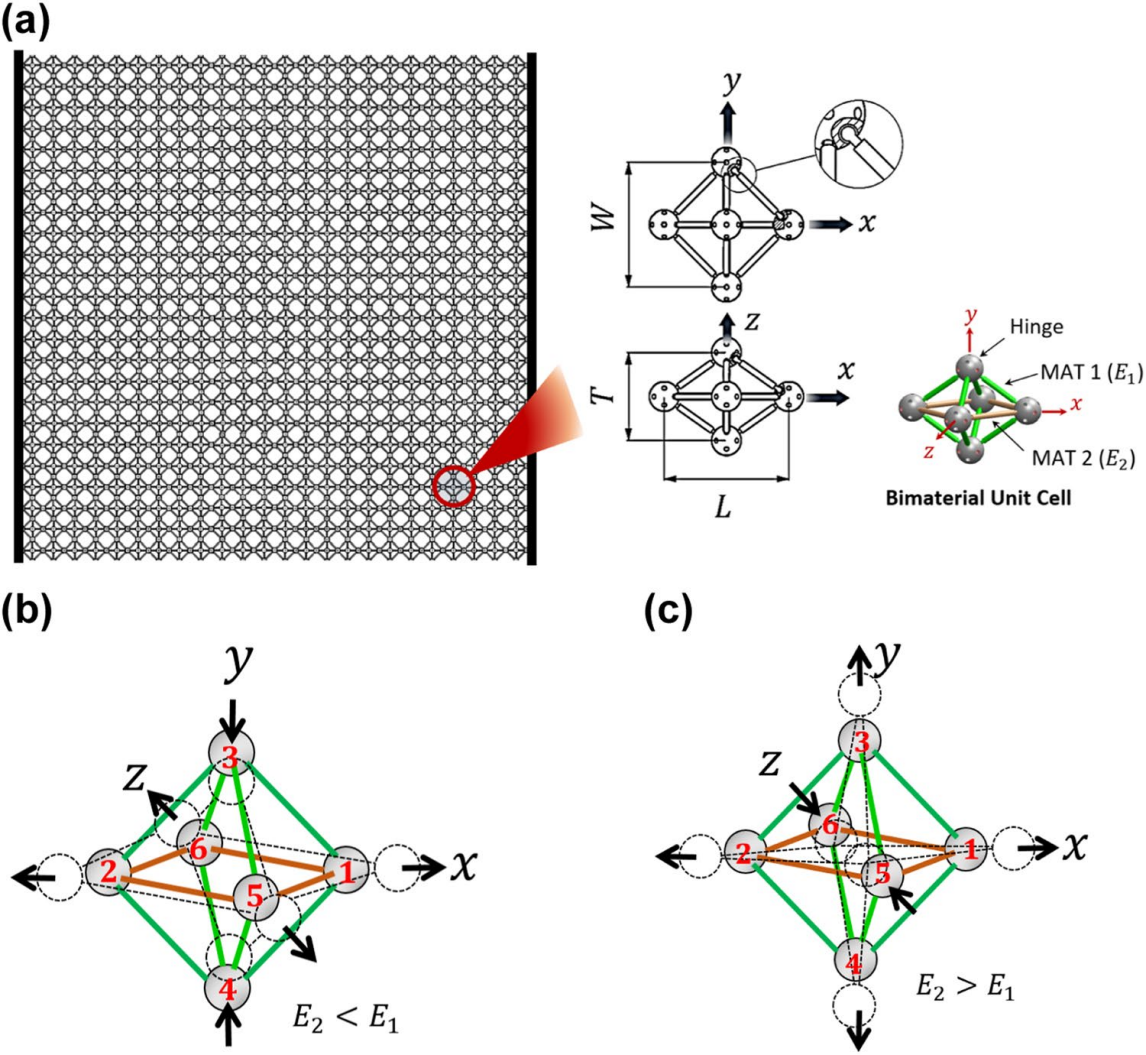
Our proposed 3D metamaterial with giant and strain-independent Poisson’s ratios. (**a**) Schematic of the proposed hinged-3D metamaterial. The unit cell is a bimaterial with struts along $xz$-plane of a distinct material (MAT 2) with Young’s modulus (${E}_{2}$). Other struts are made of MAT 1 with Young’s modulus (${E}_{1}$). All struts are hinged via specially designed spherical hinges (see Supplementary Information – S1). The characteristic geometry of the unit cell was defined by *L*, *W* and *T*, which are the extreme dimensions of the unit cell along *x*, *y*, and *z* − directions, respectively. (**b**) Schematic of the deformation of a bimaterial hinged-3D unit cell with ${E}_{2} < {E}_{1}$. Hinges 3 and 4 are pulled-in while hinges 5 and 6 are pushed out for a stretch along *x* − axis. (**c**) Schematic of the deformation of a bimaterial hinged-3D unit cell with ${E}_{2} > {E}_{1}$. Hinges 3 and 4 are pushed out while hinges 5 and 6 are pulled-in for a stretch along *x* − axis.

Our proposed hinged-3D metamaterial is shown in Fig. 1. The unit cell of this metamaterial consists of 12 hinged struts (Fig. 1(a)). Our metamaterial is a bimaterial with struts along $xz-\,$plane are made of a material with an elastic modulus ${E}_{2}$ (MAT 2) while other struts are made of a material with an elastic modulus ${E}_{1}$ (MAT 1). The struts are hinged together to allow a free rotation at their ends. The metamaterial is assembled such that hinges 1 and 2 are aligned with $x-\,$axis, hinges 3 and 4 are aligned with $y-\,$axis, and hinges 5 and 6 are aligned with $z-\,$axis, as shown in Fig. 1.

Our developed metamaterial can give Poisson’s ratios of two opposite algebraic signs when measured from two different planes. When ${E}_{2} < {E}_{1}$, the distance between hinges 3 and 4 decreases due to a unit cell stretch along $x-\,$axis (Fig. 1(b)). This indicates a positive in-plane ($xy-\,$plane) Poisson’s ratio (${\nu }_{xy}$). As long as hinges 3 and 4 approach each other, hinges 5 and 6 are pushed away indicating a negative Poisson’s ratio of $xz-\,$plane (${\nu }_{xz}$). In the case that ${E}_{2} > {E}_{1}$, the distance between hinges 5 and 6 decreases while the distance between hinges 3 and 4 increases due to a unit cell stretching along $x-\,$axis (Fig. 1(c)). For this case, ${\nu }_{xz}$ is positive while ${\nu }_{xy}$ is negative. However, both ${\nu }_{xy}$ and ${\nu }_{xz}$ are positive, when the unit cell is made of identical struts (${E}_{1}={E}_{2}$). When identical struts are used, hinges 3, 4, 5, and 6 are pulled-in equally due to a stretch along *x* – axis. This indicates that the Poisson’s ratio of the proposed metamaterial mainly depends on the contrast between ${E}_{1}$ and ${E}_{2}$.

**Figure 2:**
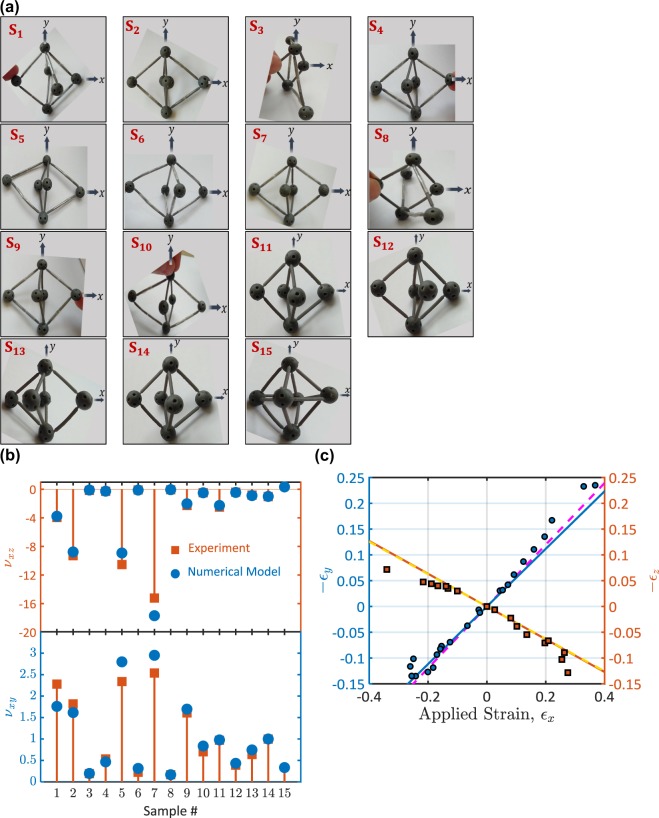
New class of hinged-3D metamaterials with giant and strain-independent Poisson’s ratios. (**a**) 3D printed and 3D-assembeled samples of unit cells with giant and constant Poisson’s ratios. The different geometry and material topology of the prepared unit cells are summarized in Table 1. (**b**) Measured Poisson’s ratios (${\nu }_{xy}$ and ${\nu }_{xz}$) of the 3D printed samples. Experimental and numerical results are compared. (**c**) The transverse strains, ${{\epsilon }}_{y}$ and ${{\epsilon }}_{z}$, as functions of the applied longitudinal strain, ${{\epsilon }}_{x}$, for a material sample with $L=29.4\,{\rm{mm}}$, $W=39.33\,{\rm{mm}}$, and $T=52.18\,{\rm{mm}}$. The experimental results (symbols) are compared to the results of the numerical model (solid lines). Curves of the linear fitting to the experimental results are represented by dashed lines.

The unit cell of the proposed metamaterial was designed to give a metamaterial with a constant Poisson’s ratio (see Supplementary Information – S1). Because of the spherical hinges, struts can freely rotate without bending. Struts are axially loaded due to a displacement in the longitudinal direction, which produces lateral displacements that depend on the unit cell geometry and the strut’s stiffness. Because struts do not exhibit bending, the transverse displacements are directly proportional to the applied longitudinal displacement. Therefore, the Poisson’s ratio is independent of the applied strain and constant for the different unit cell deformations (see Fig. 2c & *Discussion*).

To demonstrate the new class of metamaterials with giant Poisson’s ratios developed here, 15 samples of hinged-3D assembled unit cells were prepared (Fig. 2(a)). The struts and hinges were 3D printed by Stereolithography and then assembled to form a unit cell (see *Methods* & Supplementary Information – S2). The hinges were designed spherical with cut-outs to allow for the struts’ assembly (*see* Supplementary Information – S1). One unit cell was prepared with identical stiffnesses along $x$, $y$, and *z* – axes (Sample 15). This unit cell was made of identical struts (i.e., ${E}_{1}={E}_{2}$) and $L=W=T$ (where *L*, *W*, and *T* are the unit cell length, width, and thickness, respectively (see Fig. 1(a))). Other unit cell-samples (14 samples) were prepared with different struts. These unit cells were prepared with a high contrast between ${E}_{1}$ and ${E}_{2}$ (i.e., ${E}_{1}/{E}_{2}\to \infty$) and different $T/L$ and $W/L$ ratios (i.e., $T/L=0.24\to 4.21$ and $W/L=0.58\to 2.45$). The different unit cell samples are represented in Fig. 2(a). The geometries of these samples are summarized in Table 1.

**Table 1:** The geometry of the prepared samples and their measured and calculated Poisson’s ratios.

Sample #	Unit Cell Dimensions (mm)			Poisson’s Ratio (Experiment)		Poisson’s Ratio (Numerical Model)	
	L	W	T	*v*_*xy*_	*v*_*xz*_	*v*_*xy*_	*v*_*xz*_
1	52.18	39.33	26.85	2.28	−3.96	1.76	−3.78
2	52.30	38.78	17.16	1.82	−9.29	1.62	−8.77
3	17.16	38.78	52.30	0.19	−0.16	0.20	−0.11
4	26.85	39.33	52.18	0.53	−0.29	0.47	−0.27
5	66.10	39.51	22.14	2.34	−10.54	2.80	−8.91
6	22.14	39.51	66.10	0.22	−0.096	0.314	−0.112
7	67.67	39.39	16.09	2.54	−15.22	2.95	−17.69
8	16.09	39.39	67.67	0.17	−0.066	0.17	−0.057
9	59.94	46.03	42.07	1.61	−2.27	1.70	−2.03
10	42.07	46.03	59.94	0.70	−0.48	0.84	−0.49
11	33.38	33.75	22.18	0.97	−2.50	0.98	−2.265
12	22.18	33.75	33.38	0.39	−0.40	0.43	−0.44
13	29.00	33.60	30.87	0.64	−0.81	0.75	−0.88
14	31.33	31.33	31.33	0.99	−1.02	1	−1
15	31.33	31.33	31.33	0.31	0.32	0.333	0.333

*All the samples of the unit cell - except Sample #15 - were prepared such that the struts belong to the *xz* − plane were removed. This achieved a high contrast between ${E}_{1}$ and ${E}_{2}$ (i.e., ${E}_{1}/{E}_{2}\to \infty$).

The Poisson’s ratios (${\nu }_{xy}$ and ${\nu }_{xz}$) of the prepared samples were measured by means of motion analysis (*as explained in Methods*). The measured Poisson’s ratios for the different samples are represented in Fig. 2(b) and Table 1. A unit cell with identical struts (Sample 15) gives an isotropic-natural material with a positive Poisson’s ratio is the same when measured from different directions (the Poisson’s ratio of Sample 15 was determined by ${\nu }_{xy}={\nu }_{xz}\cong 1/3$). However, a metamaterial with a negative Poisson’s ratio is obtained as long as its unit cell is made of different struts (${E}_{1}\ne {E}_{2}$). For instance, the Poisson’s ratios of Sample 14 (${E}_{1}/{E}_{2}\to \infty$, $T/L=W/L=1$) were obtained equal but with opposite signs (${\nu }_{xz}\cong -\,1$ and ${\nu }_{xy}\cong 1$). The Poisson’s ratio (${\nu }_{xy}$ and/or ${\nu }_{xz}$) is obtained with a magnitude lower than one when the unit cell is made of different struts and $W/L > 1$ and/or $T/L > 1$ (see Samples 3,4,6,10-13). The smallest Poisson’s ratio values of ${\nu }_{xy}\cong 0.17$ and ${\nu }_{xz}\cong -\,0.066$ were recorded for Sample 8 where $W/L\cong 2.45$ and $T/L\cong 4.21$. In contrast, the magnitude of the Poisson’s ratio is higher than one if the metamaterial is made of a unit cell with different struts and $W/L < 1$ and/or $T/L < 1$ (see Samples 1,2,5,7,9,11). Here and for the first time, we report a hinged-3D metamaterial with constant and giant Poisson’s ratios of ${\nu }_{xz}\cong -\,16$ and ${\nu }_{xy}\cong 2.6$.

Our proposed metamaterial is of constant Poisson’s ratio that is independent of the applied strain (see Fig. 2(c)). To demonstrate this fact, a sample with $L=29.4\,{\rm{mm}}$, $W=39.33\,{\rm{mm}}$, and $T=52.18\,{\rm{mm}}$ was experimentally tested for different applied longitudinal strains, ${{\epsilon }}_{x}$. The transverse strains, ${{\epsilon }}_{y}$ and ${{\epsilon }}_{z}$, were measured for the different values of the applied strain, ${{\epsilon }}_{x}$, and plotted in Fig. 2(c). The transverse strains, ${{\epsilon }}_{y}$ and ${{\epsilon }}_{z}$, were obtained linearly varying with the applied longitudinal strain, ${{\epsilon }}_{x}$. The obtained linear variations between the transverse strains and the longitudinal strain indicate constant Poisson’s ratios, ${\nu }_{xy}=-\,{{\epsilon }}_{y}/{{\epsilon }}_{x}$ and ${\nu }_{xz}=-\,{{\epsilon }}_{z}/{{\epsilon }}_{x}$. For this sample, the Poisson’s ratios were obtained of ${\nu }_{xy}=0.59$ and ${\nu }_{xz}=-\,0.314$ by calculating the slopes of the two linear relations shown in Fig. 2(c). These linear relationships extend with excellent linearity for the considered strain range with R-squared values $> 92 \%$ and $> 97 \%$ for ${{\epsilon }}_{z}$ and ${{\epsilon }}_{y}$, respectively. The Poisson’s ratio being constant for the entire material deformation range is attributed to the utilization of the spherical hinges. Because of these spherical hinges, struts freely rotate and are stretched without bending due to the applied longitudinal strain (see *Discussions*). It should be mentioned that the struts would, however, bend/buckle when the metamaterial is deformed at a strain higher than the strain limits considered in this study. Beyond the considered strain range, the metamaterial is definitely nonlinear-elastic, and the transverse strains, ${{\epsilon }}_{y}$ and ${{\epsilon }}_{z}$, nonlinearly vary with the longitudinal strain, ${{\epsilon }}_{x}$, giving the Poisson’s ratio is strain-dependent.

**Figure 3:**
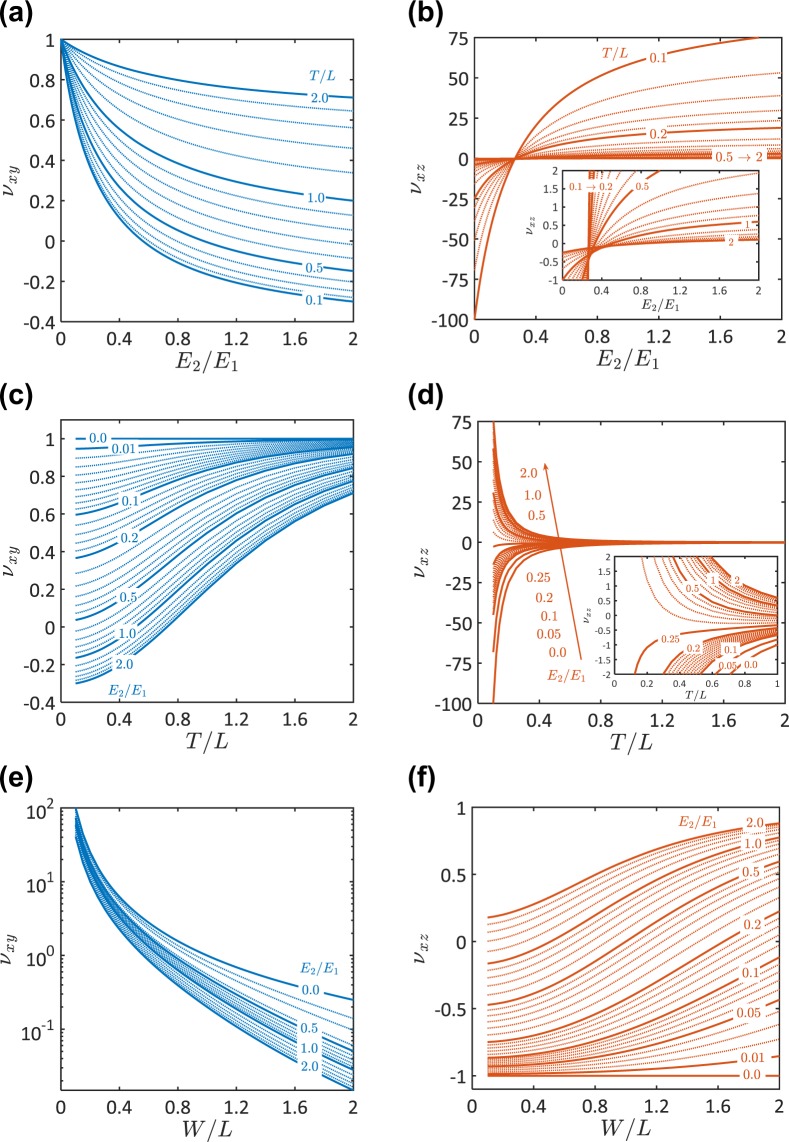
Topology effects on Poisson’s ratio of the developed bimaterial-3D metamaterial. (**a**,**b**) Influence of the contrast between ${E}_{1}$ and ${E}_{2}$ on Poisson’s ratio. (**a**) ${\nu }_{xy}$ as a function of ${E}_{2}/{E}_{1}$ ratio when $W/L=1$. (**b**) ${\nu }_{xz}$ as a function of ${E}_{2}/{E}_{1}$ ratio when $W/L=1$. (**c**,**d**,**e**,**f**) Influence of the cell geometry on Poisson’s ratio. (**c**,**e**) ${\nu }_{xy}$ as a function of $T/L$ ratio (when $W/L=1$) and $W/L$ ratio (when $T/L=1$), respectively. (**d**,**f**) ${\nu }_{xz}$ as a function of $T/L$ ratio (when $W/L=1$) and $W/L$ ratio (when $T/L=1$), respectively.

To give more insights into the role of the microstructure topology on the Poisson’s ratio of the developed metamaterial, a 3D truss numerical model was developed (see *Methods* & Supplementary Information – S3). The Poisson’s ratios of the 15 material samples were obtained by the developed 3D-truss model and compared to the experimentally determined ones in Fig. 2(b) and Table 1. In addition, the results of the proposed numerical model were compared to the results of ANSYS – a commercial numerical tool (see Supplementary Information – S4). Given the excellent agreement between our numerical and experimental results (Fig. 2), we further represent the Poisson’s ratios ${\nu }_{xy}$ and ${v}_{xz}$ as functions of ${E}_{2}/{E}_{1}$, $T/L$, and $W/L$ in Fig. 3. Results were developed for ${E}_{2}/{E}_{1}=0\to 2$, $T/L=0.1\to 2$, and $W/L=0.1\to 2$. It should be noted that the bounds of the unit cell dimensions $L$, $T$, and $W$ depend on the sizes of the struts and the spherical hinges. Struts and hinges of smaller sizes would allow for a wider range of values of the geometrical parameters $T/L$ and $W/L$. Nonetheless, using struts of small sizes would decrease the overall material stiffness and strength. Therefore, the optimum design of the metamaterial should be carried out taking into consideration the changes in the material stiffness and strength upon changing the sizes of the struts and the hinges.

An auxetic behavior was detected when a unit cell with ${E}_{2}\ne {E}_{1}$, $T\ne L$, and/or $W\ne L$ was used. An auxetic metamaterial with a negative ${\nu }_{xy}$ would be obtained if $T/L < 1$ and/or ${E}_{2}/{E}_{1} > 1$. For a unit cell with $W/L=1$, ${\nu }_{xy}$ was determined of a negative value when $T/L < 0.5$ and ${E}_{2}/{E}_{1} > 1$ (Fig. 3(a,c)). Generally, ${\nu }_{xy}$ is negative as long as $T/L\le  \sim \,0.2$ and ${E}_{2}/{E}_{1}\ge  \sim \,0.6$. On the other hand, a unit cell with $T/L < 1$, $W/L < 1$, and/or ${E}_{2}/{E}_{1} < 1$ would give an auxetic metamaterial with a negative ${\nu }_{xz}$. It follows from Fig. 3 that ${\nu }_{xz}$ is negative if $W/L\le  \sim \,0.5$ (for $T/L=1$ and ${E}_{2}/{E}_{1}=1$) or ${E}_{2}/{E}_{1}\le  \sim \,0.5$ (for $W/L=T/L=1$). In general, ${\nu }_{xz}$ is negative if ${E}_{2}/{E}_{1}\le  \sim \,0.28$ and $T/L < 1$ (Fig. 3(d)). It should be mentioned that a double negative Poisson’s ratio-metamaterial is not an option where either ${\nu }_{xy}$ or ${\nu }_{xz}$ would be positive (Fig. 3(c,d)).

It follows from Fig. 3 that the developed metamaterial is sensitive to small microstructural topology changes. For example, a slight change in ${E}_{2}/{E}_{1}$ ratio from 2 to 0.2 led to significant increase in ${\nu }_{xy}$ by $\sim 300 \%$ and decrease in ${\nu }_{xz}$ by $\sim 182 \%$ for a unit cell with $T/L=W/L=1$ (Fig. 3(a,b)). The change of Poison’s ratio is maximized by decreasing ${E}_{2}/{E}_{1}$ ratio till the lower bound limit, 0, achieving increase in ${\nu }_{xy}$ by $\sim 400 \%$ and decrease in ${\nu }_{xz}$ by $\sim 243 \%$ for a unit cell with geometrical ratios of unity. From the practical point of view, this asymptotic lower limit (${E}_{2}/{E}_{1}=0$) can be approached using a unit cell with ${E}_{2}\ll {E}_{1}$. For example, a unit cell made of metal and polymer struts would achieve ${E}_{2}/{E}_{1}\to 0$. In addition, the rate of change of the Poisson’s ratio due to a change in ${E}_{2}/{E}_{1}$ ratio increases as $T/L$ ratio decreases. For instance, ${\nu }_{xz}$ of a unit cell with $T/L=0.1$ and $W/L=1$ changed from $\sim 78$ to $\sim -\,100$ when ${E}_{2}/{E}_{1}$ ratio was decreased from 2 to 0. Results in Fig. 3(c–f) indicate that the Poisson’s ratios ${\nu }_{xy}$ and ${\nu }_{xz}$ of the developed metamaterial can be significantly altered due to a slight change in $W/L$ and $T/L$. For example, ${\nu }_{xz}$ was observed increasing from $\sim 0$ to $\sim -\,70$ due to a change in $T/L$ from 2 to 0.1 for a unit cell with ${E}_{2}/{E}_{1}=0.05$ and $W/L=1$ (Fig. 3(d)). On the other hand, ${\nu }_{xy}$ was determined increasing from $\sim 0$ to $\sim 60$ due to a change in $W/L$ from 2 to 0.1 for a unit cell with ${E}_{2}/{E}_{1}=1$ and $T/L=1$ (Fig. 3(e)).

Our proposed metamaterial exhibits a giant Poisson’s ratio if it is composed of unit cells of a high contrast between ${E}_{1}$ and ${E}_{2}$ and of $T/L\ll 1$ and/or $W/L\ll 1$ (Fig. 3(c–f)). A unit cell with $T/L=0.1$, $W/L=1$, and ${E}_{2}/{E}_{1}=0$ gave a metamaterial with a giant negative Poisson’s ratio of ${\nu }_{xz}=-\,\,100$ (Fig. 3(d)). On the other hand, a metamaterial composed of unit cells with $W/L=0.1$, $T/L=1$, and ${E}_{2}/{E}_{1}=0$ exhibited a giant positive Poisson’s ratio of ${\nu }_{xy}=100$ (Fig. 3(e)).

## Discussion

Here, we developed a new class of 3D-metamaterials that can give giant and strain-independent Poisson’s ratios. The metamaterial is composed of 3D unit cells, which are assembled from struts and spherical hinges (see Fig. 1 & Fig. S1). The material is isotropic with a positive Poisson’s ratio of $\nu =0.33$ if its unit cell is of identical stiffnesses when measured from $x$, $y$, and $z-\,$directions. However, it gives a negative Poisson’s ratio by achieving a contrast between the unit cell stiffnesses along $x$, $y$, and $z-\,$directions (see Fig. 2 & Fig. S4). The unit cell stiffness can be tailored by changing the strut’s geometry and/or material. The magnitude of the Poisson’s ratio increases as the contrast between the unit cell stiffnesses increases.

We experimentally and numerically demonstrated the microstructural topology dependence of the Poisson’s ratio of the developed metamaterial (Figs. 2 and 3). We revealed that the Poisson’s ratios, ${\nu }_{xy}$ and ${\nu }_{xz}$, robustly depend on the geometrical and material parameters of the unit cell, i.e., $T/L$, $W/L$, and ${E}_{2}/{E}_{1}$. The Poisson’s ratio would significantly decrease or increase for a slight change in $T/L$, $W/L$, and/or ${E}_{2}/{E}_{1}$. Changes in these topology parameters lead to changes in the material stiffnesses along $x$, $y$, and $z-\,$directions. An increase in the material stiffness enhances the deformation along a certain direction and inhibits the deformation along another direction. Thus, the contrast between the deformations along the different directions results in significant changes in the Poisson’s ratios. For instance, the increase in ${E}_{2}/{E}_{1}$ enhances the stiffness over the xz-plane (see Fig. 1(b,c)). As a result, the deformation along z-axis is enhanced while the deformation along y-axis is significantly inhibited. Because of this enhancement in the stiffness, ${\nu }_{xz}$ has been increased while ${\nu }_{xy}$ has been decreased due to an increase in ${E}_{2}/{E}_{1}$, as it can be seen in Fig. 3. It is not only the elastic modulus ratio that would change the stiffness but also the geometrical parameters $T/L$ and $W/L$. It was observed that ${\nu }_{xz}$ increases and ${\nu }_{xy}$ decreases upon increasing the $W/L$ ratio. This can be attributed to the decrease in the material stiffness along yz-plane due to an increase in the $W/L$ ratio (i.e., the stiffness of a truss element decreases as its length increases). The increase in the $T/L$ ratio decreases the material stiffness along xz- and yz-planes. This, in turn, enhances the Poisson’s ratio over xy-plane (${\nu }_{xy}$), while the Poisson’s ratio over xz-plane (${\nu }_{xz}$) would increase/decrease depending on the other parameters.

We experimentally demonstrated a giant negative Poisson’s ratio of −16 (Sample 7). This giant Poisson’s ratio has been achieved using a unit cell of dissimilar materials and stiffnesses. The significant dissimilarity achieved in the unit cell stiffnesses resulted in a huge anisotropy, which is the main cause behind the achieved giant Poisson’s ratio. According to Zener’s anisotropy ratio, $A=2{C}_{44}/({C}_{11}-{C}_{12})$, and the Poisson’s ratio-stiffness relation, $\nu ={C}_{12}/({C}_{11}+{C}_{12})$, of cubic crystals, the Poisson’s ratio is maximum of $\nu \to 1/2$ if the crystal is highly anisotropic with $A\to \infty$ and ${C}_{11}={C}_{12}$. In addition, early investigations on the Poisson’s ratio recommended highly anisotropic materials to give large negative Poisson’s ratios^22,24^. It was demonstrated that 69% of cubic crystals have negative and giant Poisson’s ratios when stretched along [110] direction. This has been attributed to the anisotropic nature of BCC and FCC crystals. The Poisson’s ratios of a cubic crystal measured from the transverse directions to [110] direction are maxima of −1 and 2 if the crystal is highly anisotropic with $A\to \infty$ and ${C}_{11}={C}_{12}$. This has been achieved via our proposed design of the metamaterial where the material anisotropy is promoted by tailoring the unit cell geometry and/or using dissimilar materials.

In our developed 3D metamaterial, the giant negative Poisson’s ratio relies on microstrain fields at the unit cell, which produce because of the struts’ stretching. Previous studies demonstrated that giant Poisson’s ratios of magnitudes higher than 1 can be achieved by promoting microscopic fields, e.g., microstrains or microrotations. For example, the giant Poisson’s ratio of cellular materials has been attributed to flexural micro-deformations^35^. In addition, a giant Poisson’s ratio of −12 was determined for microporous foams, which has been attributed to micro-rotation fields^22^. Furthermore, the theoretical mechanics using Cosserat and micromorphic models demonstrated that metamaterials with exotic properties can be produced as long as their microstructures are designed to promote microscopic rotations or deformations^2,36^. These microscopic fields promote the transverse displacement for the material’s longitudinal deformation, and hence lead to giant negative Poisson’s ratios^36^.

Unlike existing designs of metamaterials^22,27–29,32,33^, our proposed metamaterial is of constant Poisson’s ratios. In our metamaterial, the transverse displacement is directly proportional to the applied longitudinal displacement. Because of the spherical hinges, struts freely rotate and are axially stretched without bending due to a displacement in the longitudinal direction. This design has led to a constant Poisson’s ratio that is independent of the applied strain (Fig. 2(c)). In contrast, existing designs of metamaterials secrete Poisson’s ratios that strongly depend on the applied strain. The Poisson’s ratio of microporous foam is giant of −12 only when the material is subjected to a longitudinal strain of 15%, and the Poisson’s ratio value decreases for other strains different than 15%^22^. In addition, the Poisson’s ratio of carbon nanotube-yarns increased from 3.3 to 4.2 due an increase in the tensile strain from 5% to 9%^27^. In a recent study, a multi-material 3D metamaterial exhibited different negative Poisson’s ratios when changing the applied strain between 3% to 18%^32^. These different designs secrete Poisson’s ratios that depend on the applied strain because the unit cell of the metamaterial exhibits a nonlinear relation between the longitudinal and transverse displacements. This nonlinearity produces due to flexural bending of the fibrils or struts of the unit cell. Unlike these designs, we implement spherical hinges, which avoid the struts’ bending; therefore, the longitudinal and transverse displacements are linearly related and the Poisson’s ratio is constant for the entire material deformation range.

In addition to the promising applications of auxetic metamaterials, which include the development of advanced sensors and actuators, our developed metamaterial would replace existing materials used for making strain amplifiers and strain gauges. A metamaterial with a Poisson’s ratio higher than 1 gives a good strain amplifier with an amplification factor bigger than −1. Our metamaterial gives a linear relation between longitudinal and lateral strains and gives the amplification factor constant and equal to −*ν*.

## Methods

### 3D printing and samples preparation

15 samples of hinged-3D assembled unit cells were prepared to demonstrate the giant Poisson’s ratio of our developed metamaterial (Fig. 2(a)). The unit cell is composed of struts connected via spherical hinges (see Figs. 1 and 2 & Supplementary Information – S1). The struts and hinges were 3D printed by Stereolithography (SLA) of Photoreactive polymer resin (FLGPGR04, density 1.09 ${\rm{g}}/{{\rm{cm}}}^{3}$, viscosity 900 cps) provided by Formlabs, Inc. A Stereolithography 3D printer (Form Labs-Form2) with 25 μm layer thickness resolution and 140 μm laser spot diameter was used (see Supplementary Information – S2). Bottom-top building procedure was followed during the printing process. After printing, parts were rinsed in isopropyl alcohol (IPA) to remove uncured resin from the parts’ surface. Parts were post-cured by exposure to ultraviolet light (using UV Nail Salon (see Fig. S2)) for 30 min. Afterwards, the interface-surfaces of the struts and hinges were ground using a series of fine sandpapers of 2000–4000 grit size. Then, struts and hinges were assembled to form a 3D-hinged unit cell.

### Poisson’s ratio measurements

The Poisson’s ratio of each of the prepared 15 samples of unit cells were measured by means of video and image analyses. The deformation of the unit cell was video recorded using a high resolution camera. Then, an image correlation software (*ImageJ*) was used to measure the unit cell deformation along $x,y,$ and *z* – directions. The Poisson’s ratio was calculated based on the measured deformations, as follows:

1 $$\begin{array}{c}{\nu }_{xy}=-\,\frac{{\varepsilon }_{y}}{{\varepsilon }_{x}}=-\,\frac{\Delta y\,L}{\Delta x\,W}\\ \,{\nu }_{xz}=-\,\frac{{\varepsilon }_{z}}{{\varepsilon }_{x}}=-\,\frac{\Delta z\,L}{\Delta x\,T}\end{array}$$

where $\Delta x$, $\Delta y$, and $\Delta z$ are the deformations of the unit cell along $x,y,$ and $z-\,$directions, respectively. The deformations $\Delta x$, $\Delta y$, and $\Delta z$ were determined as the average of 7 measurements. *L*, *W*, and *T* are the unit cell length, width, and thickness, respectively.

It should be noted that the Poisson’s ratios, ${\nu }_{xy}$ and ${\nu }_{xz}$, are constants if the longitudinal displacement $\Delta x$ is linearly proportional to the transverse displacements $\Delta y$ and $\Delta z$, i.e., $\Delta y=a\Delta x$ and $\Delta z=b\Delta x$ where *a* and *b* are two constants represent the slopes of the linear relations. The substitution of these relations into Eq. (1) gives:

2 $${\nu }_{xy}=-\,aL/W\,{\rm{and}}\,{\nu }_{xz}=-bL/T$$

It is clear that the Poisson’s ratios are constants as long as the transverse displacements are proportional and linearly vary with the longitudinal displacements.

### Numerical model

To investigate the microstructural topology effects on the Poisson’s ratio of the developed hinged-3D metamaterial (Fig. 3), a 3D truss model was developed. The 3D metamaterial is discretized such that each of the struts is a two-nodes bar element that exhibits 6 degrees of freedom in the global-3D domain. The elastostatic equilibrium of the 3D metamaterial was expressed in the matrix form, as follows:

3 $${\boldsymbol{KU}}={\boldsymbol{F}}$$

where ${\boldsymbol{F}}$ is the applied force vector, and ${\boldsymbol{U}}$ is the nodal displacements global vector. ${\boldsymbol{K}}$ is the global stiffness matrix, which was defined as follows:

4 $${\boldsymbol{K}}=\mathop{\sum }\limits_{n=1}^{{N}_{e}}{{\boldsymbol{C}}}^{T}{\boldsymbol{kC}}\,{\rm{with}}\,{\boldsymbol{k}}={{\boldsymbol{T}}}^{T}{\boldsymbol{DT}}$$

where ${N}_{e}$ is the total number of struts that form the metamaterial. ${\boldsymbol{C}}$ is the node connection matrix which maps the elements’ stiffness matrix into the global matrix. $${\boldsymbol{D}}=\frac{EA}{{L}_{e}}[\begin{array}{cc}1 & -1\\ -1 & 1\end{array}]$$ is the strut’s local stiffness matrix. ${\boldsymbol{T}}$ is a transformation matrix, which was defined as follows:

5 $${\boldsymbol{T}}=\frac{1}{{L}_{e}}[\begin{array}{cccccc}{x}_{j}-{x}_{i} & {y}_{j}-{y}_{i} & {z}_{j}-{z}_{i} & 0 & 0 & 0\\ 0 & 0 & 0 & {x}_{j}-{x}_{i} & {y}_{j}-{y}_{i} & {z}_{j}-{z}_{i}\end{array}]\,$$

where ${L}_{e}$ is the strut’s length. $({x}_{i},{y}_{i},{z}_{i})$ and $({x}_{j},{y}_{j},{z}_{j})$ are the coordinates of the ends of a strut-element in the 3D domain. *E* and *A* are the Young’s modulus and cross-sectional area of the strut.

According to Eqs. (3) and (4), the element stifness matrix, ${\boldsymbol{k}}$, in the 3D space was determined in the form:

6 $${\boldsymbol{k}}=\frac{EA}{{L}_{e}}[\begin{array}{cccccc}{({x}_{j}-{x}_{i})}^{2} & ({x}_{j}-{x}_{i})({y}_{j}-{y}_{i}) & ({x}_{j}-{x}_{i})({z}_{j}-{z}_{i}) & -{({x}_{j}-{x}_{i})}^{2} & -({x}_{j}-{x}_{i})({y}_{j}-{y}_{i}) & -({x}_{j}-{x}_{i})({z}_{j}-{z}_{i})\\ ({x}_{j}-{x}_{i})({y}_{j}-{y}_{i}) & {({y}_{j}-{y}_{i})}^{2} & ({y}_{j}-{y}_{i})({z}_{j}-{z}_{i}) & -({x}_{j}-{x}_{i})({y}_{j}-{y}_{i}) & -{({y}_{j}-{y}_{i})}^{2} & ({y}_{j}-{y}_{i})({z}_{j}-{z}_{i})\\ ({x}_{j}-{x}_{i})({z}_{j}-{z}_{i}) & ({y}_{j}-{y}_{i})({z}_{j}-{z}_{i}) & {({z}_{j}-{z}_{i})}^{2} & -({x}_{j}-{x}_{i})({z}_{j}-{z}_{i}) & -({y}_{j}-{y}_{i})({z}_{j}-{z}_{i}) & -{({z}_{j}-{z}_{i})}^{2}\\ -{({x}_{j}-{x}_{i})}^{2} & -({x}_{j}-{x}_{i})({y}_{j}-{y}_{i}) & -({x}_{j}-{x}_{i})({z}_{j}-{z}_{i}) & {({x}_{j}-{x}_{i})}^{2} & ({x}_{j}-{x}_{i})({y}_{j}-{y}_{i}) & ({x}_{j}-{x}_{i})({z}_{j}-{z}_{i})\\ -({x}_{j}-{x}_{i})({y}_{j}-{y}_{i}) & -{({y}_{j}-{y}_{i})}^{2} & -({y}_{j}-{y}_{i})({z}_{j}-{z}_{i}) & ({x}_{j}-{x}_{i})({y}_{j}-{y}_{i}) & {({y}_{j}-{y}_{i})}^{2} & ({y}_{j}-{y}_{i})({z}_{j}-{z}_{i})\\ -({x}_{j}-{x}_{i})({z}_{j}-{z}_{i}) & ({y}_{j}-{y}_{i})({z}_{j}-{z}_{i}) & -{({z}_{j}-{z}_{i})}^{2} & ({x}_{j}-{x}_{i})({z}_{j}-{z}_{i}) & ({y}_{j}-{y}_{i})({z}_{j}-{z}_{i}) & {({z}_{j}-{z}_{i})}^{2}\end{array}]$$

### 3D domain discretization and FE implementation

A 3D elastic domain was considered to represent our proposed metamaterial with 3D assembled unit cells. The elastic domain was discretized into bar elements with 3D-hinged ends representing the struts (see Supplementary Information – S3). A distributed load was applied acting on a surface whose normal is *x* − axis, and the opposite surface was free to move only along y and *z* – axes. The other surfaces were free surfaces. The details of the discretization scheme are found in Supplementary Information–S3. The Poisson’s ratio was calculated using Eq. (1) where the average displacements along $x,y,z-\,$axes ($\Delta x$, $\Delta y$, and $\Delta z$) were determined. The developed numerical model was verified by a comparison to ANSYS (see Supplementary Information – S4).

## Data Availability

The data that support the findings of this study are included in the article and Supplementary Information.

## Supplementary infromation

Supplementary infromation.
