# Supplementary material for: Hinged-3D metamaterials with giant and strain-independent Poisson’s ratios
Source: Sci Rep. 2020 Feb 10;10:2228. doi: 10.1038/s41598-020-59205-x (PMC7010739; doi:10.1038/s41598-020-59205-x)
Supplement: Supplementary file 1 — Supplementary infromation. [file 41598_2020_59205_MOESM1_ESM.pdf]

Supplementary Information

Hinged-3D metamaterials with giant and strain-independent Poisson's ratios

Mohamed Shaat[1*] & Ahmed Wagih[2]

[1]Mechanical Engineering Department, Abu Dhabi University, Al Ain, P.O.BOX 1790, United Arab Emirates.
[2]Department of Mechanical Engineering, Zagazig University, Zagazig 44511, Egypt.

Contents

S1: Unit Cell Design

S2: Stereolithography 3D Printing

S3: Finite Element Modeling (Our Developed Numerical Model)

S4: Numerical Simulations using ANSYS

References

S1: Unit Cell Design

Inspired by BCC crystals, which have shown a giant Poisson's ratio $-1 \leq \nu \leq 2$[1], we designed a metamaterial with hinged-3D assembled unit cells that can give a giant and strain-independent Poisson's ratios. The unit cell is composed of 6 spherical hinges and 12 struts, as shown in Fig.1 and Fig.S1. The spherical hinge was specially designed to give a free rotation of the struts. 12 spherical cut-outs with 2.1 mm diameter were cut and distributed on the circumstance of the spherical hinge to connect the struts. The end of the strut was a spherical cap of 2 mm diameter to fit into the spherical cut-out of the hinge (Fig.S1(a)). The spherical cut-outs were

---

Correspondence and requests for materials should be addressed to M. Shaat

Email: mohamed.i@adu.ac.ae ; shaatscience@yahoo.com

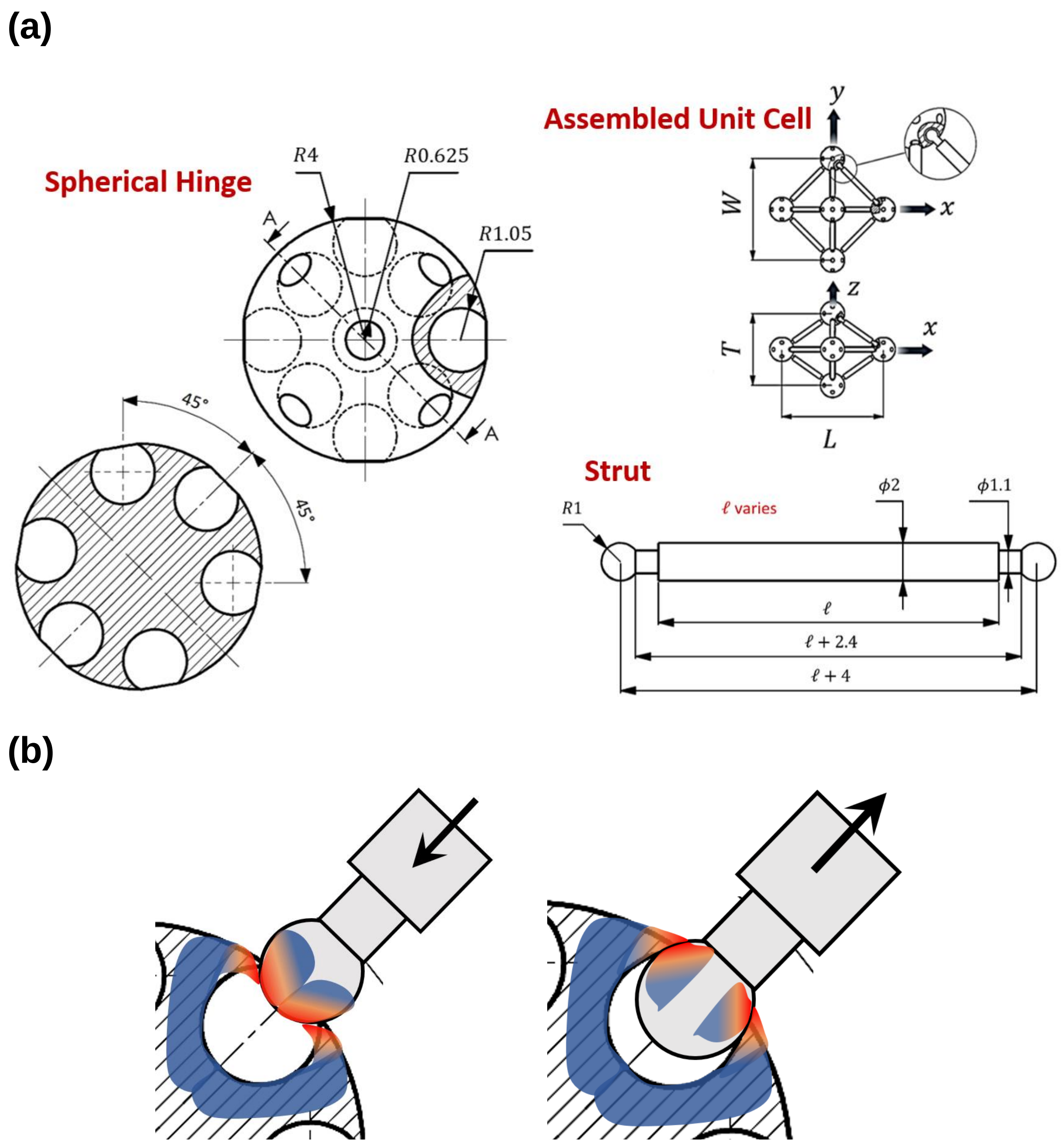

Figure S1: Detailed geometries of the unit cell of the proposed 3D metamaterial. (a) Dimensions of the spherical hinge and the strut (*all dimensions in* mm). The length of the struts varies depending on the cell geometry (see Table1). (b) A schematic of the deformation of the spherical hinge at the cut-out when the strut is assembled (left) and tensile loaded (right).

designed with an external opening of 1.25 mm diameter to avoid a separation of the strut while it is loaded. A neck of 1.1 mm diameter and 2.4 mm length was shaped between the strut body and

its spherical end to permit the strut's rotation during the unit cell deformation. The dimensions of the spherical hinge and the strut were carefully chosen such that the struts can be easily assembled to the spherical hinges with no separation of stretched struts during the unit cell deformation (see Fig. S1).

S2: Stereolithography 3D Printing

The struts and spherical hinges were 3D printed by Stereolithography (SLA) printing technique available at 3D-layers©. Fig. S2 shows the 3D printing process of the spherical hinges and struts. The process started with a computer aided design (CAD) of the parts to be printed using *SolidWorks*. Then, parts were assembled and checked for interference using SolidWorks' interference check tool. Afterwards, the CAD parts were exported into STL files. The dimensions of the parts to be printed and their printability were verified using a 3D builder tool (Fig. S2(a)). A photoreactive polymer resin (FLGPGR04) was used (Fig.S2(c)). Bottom-up building technique with 205 layers was applied to build the spherical hinges while 65 layers was applied to build up the struts. A solid base with pin-shaped two supports was built first from the same resin material to allow the building of the spherical hinge since it is not possible to start building a spherical geometry from a point (Fig.S2(b)). In addition, the strut was supported horizontally with 6 supports such that two supports were used underneath the spherical ends and the other four were distributed over the struts body to avoid bending while printing (Fig.S2(b)). The temperature of the resin was kept constant at 30°C and the processing time for a single strut was 30 min while for one spherical hinge was 60 min. After printing, the parts were submerged in isopropyl alcohol (IPA) to remove any contaminants of uncured resin. Then, the base and supports were removed of the parts. Afterwards, parts were subjected to ultraviolet light for 30 min (Fig.S2(c)). Finally, the spherical cap-ends of the strut were polished using a series of sandpapers up to 4000 grit size.

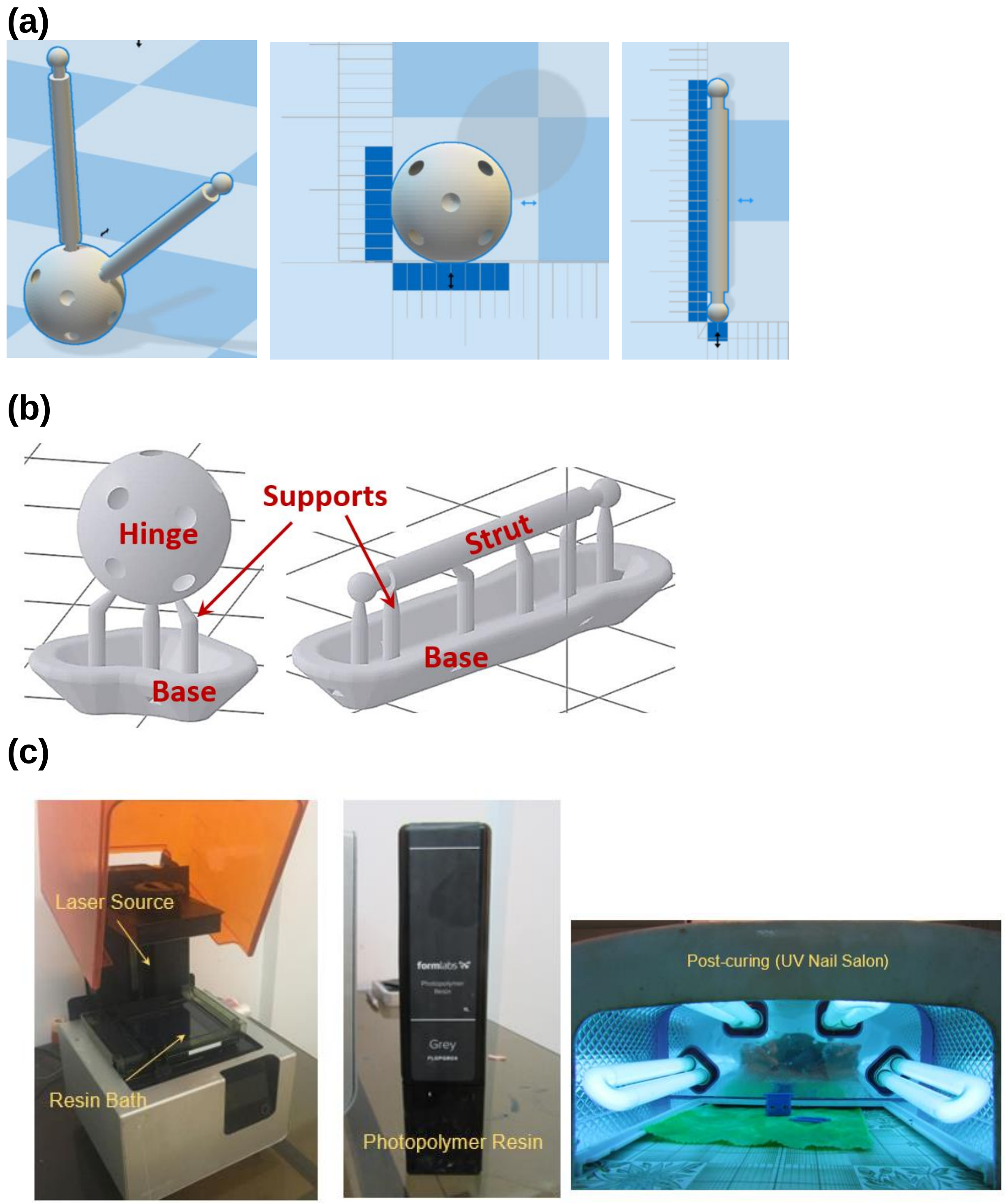

Figure S2: Stereolithography (SLA) 3D-Printing Process. (a) Dimensions verification and printability check. (b) Supporting struts and spherical hinges by building a base and pin-shaped supports. (c) Stereolithography 3D printing equipment. The Form Labs-Form2 printer (left), Photopolymer Resin (FLGPGR04) (middle), and an ultraviolet (UV) Nail Salon for post-curing of 3D printed parts.

S3: Finite Element Modeling (Our Developed Numerical Model)

A computational package of a 3D-truss finite element model was built to investigate the microstructural topology effects on the Poisson's ratio and to extract the results presented in Fig. 3. Three main parameters affect the Poisson's ratio, e.g. T/L, W/L, and E_2/E_1. We found that investigating the influence of each one of these parameters using a commercial software, e.g. ANSYS, is time consuming. Therefore, we built our own 3D-truss model of the designed metamaterial, which can effectively give results in a short time.

A discretization procedure was utilized where nodes and their coordinates were defined over x, y, and z-directions as shown in Fig. S3. Then, elements were setup between these nodes where elements' numbers were assigned, and nodes were specified for each element. In addition, the cross sectional area and Young's modulus were defined for each element. These data were automatically generated using the discretization procedure and saved in excel files. The outputs of the discretization approach were used to form the element stiffness matrix of all elements, and then the global element stiffness matrix. Boundary conditions were then defined such that a distributed load was applied acting on a surface whose normal is $x-$axis, and the opposite surface was free to move only along y and $z-$axes (Fig. S3). The other surfaces were free surfaces allowed to move in x, y, and z-directions.

S4: Numerical Simulations using ANSYS

3D-truss elements were used to build our proposed metamaterial model using ANSYS commercial software to simulate the predicted Poisson's ratio of a single cell and a structure made of the proposed metamaterial. The model was built to using LINK180 elements with circular cross section and a predefined diameter of 1 mm. Two materials were defined using elastic material model in the library of the software. For all the tested materials (except sample 15), a material with a low Young's modulus of $E_2 = 0.001$ GPa was assigned for links located in the $xy-$plane

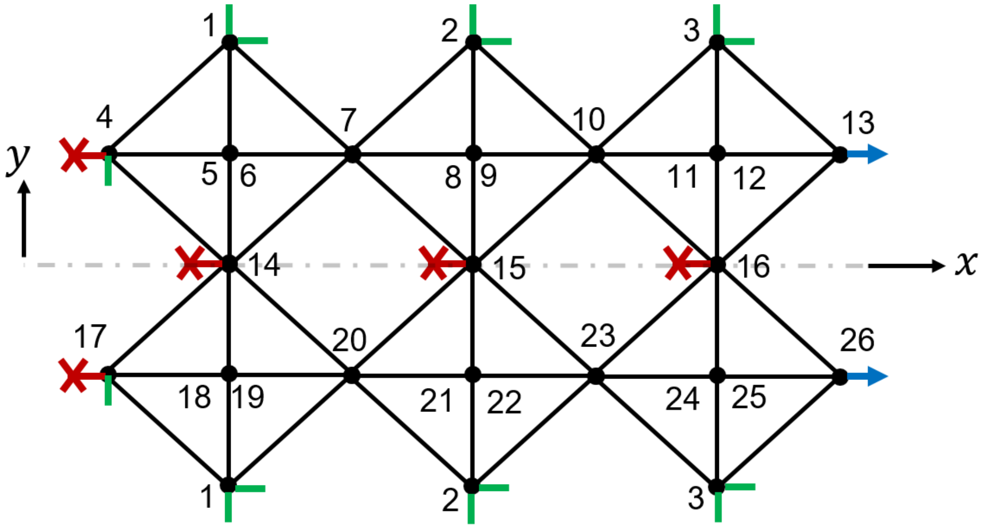

Figure S3: Finite element modeling of the proposed metamaterial. An example of a metamaterial discretization along xy-plane. Node numbering is depicted along with boundary conditions. All nodes are free to move along x, y, and z-directions unless else is defined by green and red indicators.

while steel with a Young's of $E_1 = 210\ \mathrm{GPa}$ was assigned for other links. These chosen elastic modulus values achieve a stiffness ratio as high as $E_1/E_2 = 2.1 \times 10^4 \rightarrow \infty$. For sample 15, all links were modeled of the same materials with a Young's modulus of $E_2 = E_1 = 210\ \mathrm{GPa}$.

Simulations were performed to determine the Poisson's ratios of the 15 material samples. The Poisson's ratio as determined by ANSYS are compared to the ones determined by our developed finite element model (Section S3) in Table S1. All of these results are also compared to the experimentally determined ones (Table S1). Table S1 shows an excellent match between the results of ANSYS and our proposed finite element model. This demonstrates the effectiveness of our developed numerical model and the validity of the results presented in Fig. 3. In addition, an excellent match between the numerical results and the experimental measurements of the Poisson's ratio is very clear in Table S1. This demonstrates the effectiveness of our designed spherical hinges, which gave a free rotation with a nearly-zero friction between the struts and hinges.

Table S1: Comparison between ANSYS, our proposed numerical model, and experiment. Poisson's ratios of the processed samples as determined by the three methods.

	Unit Cell Dimensions (mm)			Poisson's Ratio					
				Experiment		Our Numerical Model		ANSYS FE	
Sample #	L	W	T	ν_{xy}	ν_{xz}	ν_{xy}	ν_{xz}	ν_{xy}	ν_{xz}
1	52.18	39.33	26.85	2.28	-3.96	1.76	-3.78	1.76	-3.76
2	52.30	38.78	17.16	1.82	-9.29	1.62	-8.77	1.82	-9.29
3	17.16	38.78	52.30	0.19	-0.16	0.20	-0.11	0.19	-0.11
4	26.85	39.33	52.18	0.53	-0.29	0.47	-0.27	0.47	-0.27
5	66.10	39.51	22.14	2.34	-10.54	2.80	-8.91	2.79	-8.91
6	22.14	39.51	66.10	0.22	-0.096	0.314	-0.112	0.31	-0.11
7	67.67	39.39	16.09	2.54	-15.22	2.95	-17.69	2.87	-17.68
8	16.09	39.39	67.67	0.17	-0.066	0.17	-0.057	0.17	-0.057
9	59.94	46.03	42.07	1.61	-2.27	1.70	-2.03	1.69	-2.029
10	42.07	46.03	59.94	0.70	-0.48	0.84	-0.49	0.83	-0.49
11	33.38	33.75	22.18	0.97	-2.50	0.98	-2.265	0.98	-2.26
12	22.18	33.75	33.38	0.39	-0.40	0.43	-0.44	0.43	-0.44
13	29.00	33.60	30.87	0.64	-0.81	0.75	-0.88	0.74	-0.88
14	31.33	31.33	31.33	0.99	-1.02	1	-1	1	-1
15	31.33	31.33	31.33	0.31	0.32	0.333	0.333	0.33	0.33

Fig.S4 shows the deformed shape of materials modeled using unit cells, samples 7 and 15, in two different planes to highlight the difference between the deformation of an isotropic material and our proposed metamaterial. As shown in the figure, when a tensile load was applied in x-direction, the nodes in y-direction were pulled in for both samples showing positive Poisson's ratio ν_{xy}. In contrast, the nodes in z-direction were pushed-out just for Sample 7 giving a negative Poisson's ratio ν_{xz}. As for Sample 15, nodes along z-direction were pulled in showing a positive Poisson's ratio ν_{xz}. This demonstrates that our proposed metamaterial achieves a negative Poisson's ratio if the material anisotropy is promoted (see *Discussions* in the paper).

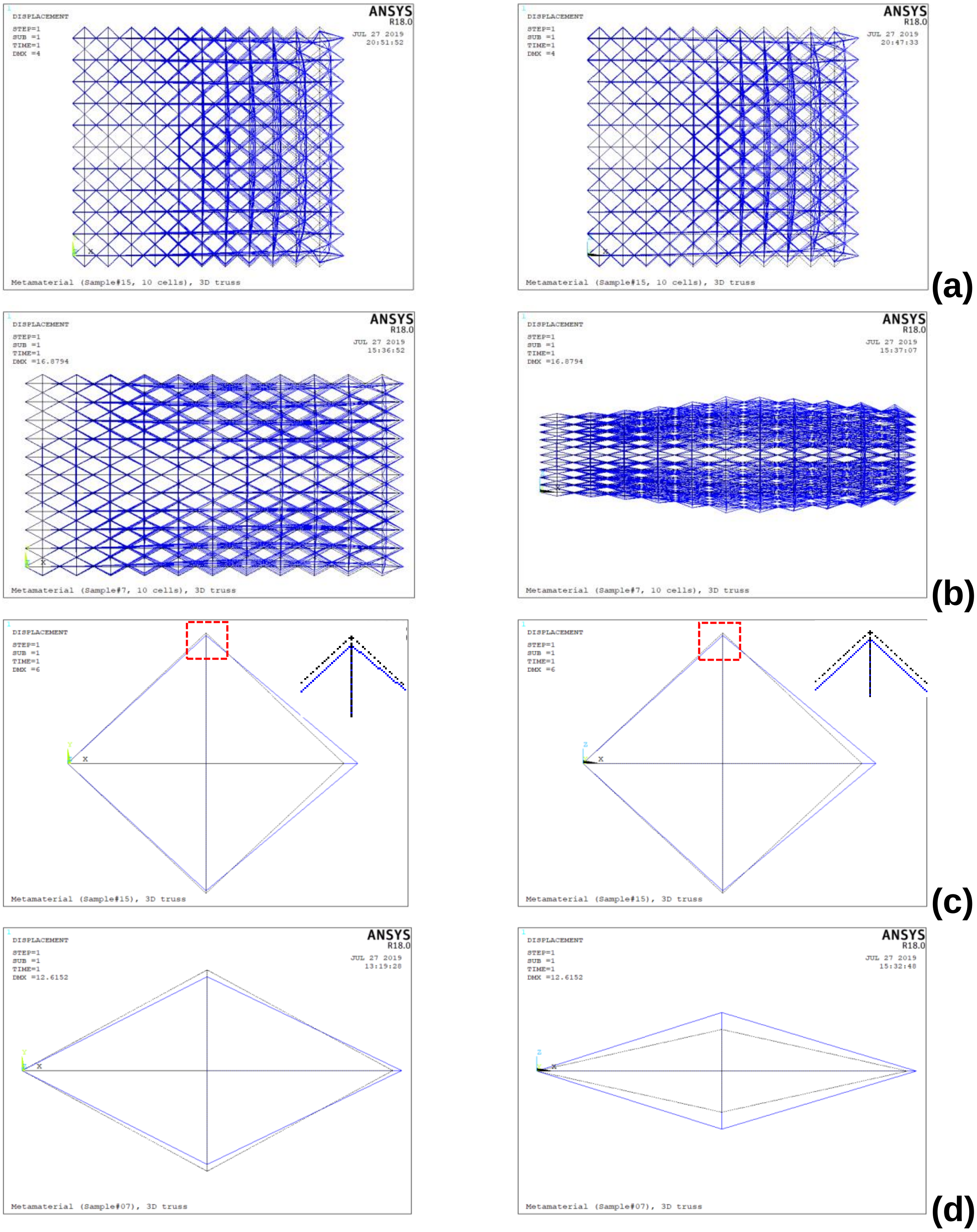

Figure S4: Deformation of a bimaterial 3D-metamaterial with large anisotropy and a negative Poisson's ratio (Sample 7) in comparison to the deformation of an isotropic material (Sample 15). (a) Deformation of an isotropic material with a unit cell of identical stiffnesses along x, y, and z –directions (Sample 15). The material is modeled with 1000 unit cells

$(10 \times 10 \times 10)$. Positive Poisson's ratios ν_{xy} and ν_{xz} were obtained for this isotropic material. The deformation of a single unit cell is shown in (c). (b) Deformation of a metamaterial with large anisotropy. The anisotropy has been achieved by using a unit cell of dissimilar materials (Sample 7). The material is modeled with 1000 unit cells $(10 \times 10 \times 10)$. A positive Poisson's ratios ν_{xy} was obtained while a negative Poisson's ratio ν_{xz} was observed. The deformation of a single unit cell is shown in (d).

References

1. Baughman, R. H., Shacklette, J. M., Zakhidov, A. A. & Stafstro, S. Negative Poisson's ratios as a common feature of cubic metals. *Nature* **392**, 362–365 (1998).
